# Which orally administered antithrombotic agent is most effective for preventing venous thromboembolism after total knee arthroplasty? A propensity score-matching analysis

**DOI:** 10.1186/s43019-021-00093-4

**Published:** 2021-03-20

**Authors:** Seonpyo Jang, Woo Cheol Shin, Min Ku Song, Hyuk-Soo Han, Myung Chul Lee, Du Hyun Ro

**Affiliations:** 1 Department of Orthopedic Surgery, Seoul National University Hospital, 101 Daehak-ro, Jongno-gu, Seoul, 110-744 South Korea; 2 Seoul National University College of Medicine, Seoul, South Korea; 3 Department of Orthopedic Surgery, Jounachim Hospital, Guri, South Korea

**Keywords:** Venous thromboembolism, Total knee arthroplasty, Antithrombotic agent, Aspirin, Rivaroxaban, Apixaban

## Abstract

**Purpose:**

Even today, total knee arthroplasty (TKA) is associated with venous thromboembolism (VTE). The purpose of our study is to report the incidence of postoperative VTE and to compare the efficacy of commonly used orally administered antithrombotic agents.

**Materials and methods:**

Seven hundred ad ninety-nine patients who underwent primary TKA were retrospectively reviewed. The patients were prescribed one of three antithrombotic agents: aspirin (*n* = 168), rivaroxaban (*n* = 117), or apixaban (*n* = 514). Before surgery, patient demographics and risk factors were matched via propensity scoring. After surgery, all three groups took the agent for 7 days and underwent ultrasonography to check for VTE.

**Results:**

The overall incidence of postoperative VTE was 15.4% (123/799). Only one patient developed symptomatic VTE. Female sex and staged bilateral TKA were risk factors for postoperative VTE. The postoperative VTE rates in the aspirin, rivaroxaban, and apixaban groups were 16.2%, 6.0%, and 17.1%, respectively, significantly lower in the rivaroxaban group (*p* <  0.02). The majority of VTEs in all three groups were calf-vein thromboses.

**Conclusions:**

All agents showed enough efficacy as antithrombotic agents. Considering that aspirin is inexpensive, aspirin is a cost-effective option for preventing postoperative VTE.

## Introduction

Total knee arthroplasty (TKA) is effective for treating advanced degenerative arthritis of the knee. Patients undergoing artificial joint surgery have an average of two to three comorbidities and 33% have five or more [1, 2], increasing the risk for complications after surgery [3]. Given the increased numbers of artificial joint surgeries in elderly patients, the numbers of patients with comorbidities will likely increase [4], as will the rate of complications. Of these, venous thromboembolism (VTE) is a principal cause of mortality in the 3 to 6 months after surgery, and may progress to a pulmonary embolism [5]. The incidence of VTE after TKA varies from 1.5% to 41.7% [6]. Common risk factors for VTE include a previous history of VTE, venous insufficiency, congenital heart failure, obesity, trauma, immobilization, and infectious disease [3, 7]. Prevention of postoperative VTE requires significant resources [7].

Mechanical devices and pharmacological agents are used to this end. Antithrombotic agents include enoxaparin, aspirin, rivaroxaban, and apixaban [8–10]. Enoxaparin is a low-molecular-weight heparin (LMWH). The American College of Clinical Pharmacy (ACCP) guidelines recommended LMWH as an antithrombotic agent in orthopedic surgery [11]. However, it is uncomfortable for patients because enoxaparin is taken by subcutaneous injection. Aspirin is an antiplatelet agent that irreversibly inhibits the cyclooxygenase enzyme. Aspirin is an inexpensive oral agent and is recommend as an antithrombotic agent to prevent VTE in various guidelines [11, 12]. Its use is associated with a postoperative VTE frequency of 2.3–17.8% [8]. Apixaban is a direct-acting orally administered anticoagulant (DOAC), a reversible direct inhibitor of both free and clot-bound factor Xa. The ACCP guidelines recommend apixaban as an antithrombotic agent in arthroplasty surgery [11]. Its use is associated with a postoperative VTE frequency of 0.7–9% [10, 13]. Rivaroxaban is another factor Xa inhibitor (a DOAC) associated with a postoperative VTE frequency of 0.7–3.7% [14, 15]. Like apixaban, it is recommended as an antithrombotic agent in arthroplasty surgery [11].

DOACs are widely used antithrombotic agents; many reports on drug efficacies and safety have appeared. Compared to enoxaparin (a low-molecular-weight heparin (LMWH) widely used in the past), DOACs are similarly safe (associated with low bleeding risks) but more effective [16]. Apixaban is the DOAC with the lowest bleeding risk, but with no loss of efficacy [17]. Aspirin is safest in patients with postoperative anemia or who require transfusions, being better than LMWH but less effective than DOACs [18].

Previous studies on antithrombotic agents preventing VTE after TKA have compared LMWH and DOACs, or various DOACs. However, the most commonly used orally administered antithrombotic agents (aspirin, rivaroxaban, and apixaban) have not been compared. Large-scale studies are required to explore the incidence of VTE in patients taking antithrombotic agents, and the efficacies of the various drugs.

The goals of this study were: (1) to report the incidence of postoperative VTE and the associated risk factors, and (2) to compare the efficacy of three commonly used orally administered antithrombotic agents.

## Materials and methods

### Study subjects

The study was performed as a retrospective review using propensity score-matching. From January 2014 to May 2019, patients scheduled for primary TKA to treat degenerative knee arthritis were enrolled after providing informed consent and were prescribed one of three orally administered antithrombotic agents depending on the period. (From January 2014 to July 2014: rivaroxaban (Xarelto; Bayer, Germany), From July 2014 to July 2017: apixaban (Eliquis; BMS/Pfizer, New York, NY, USA), From November 2017 to December 2018: aspirin (Astrix; Boryung, Korea). Initially, 857 patients were enrolled. The exclusion criteria were simultaneous bilateral TKA (*n* = 6) and preoperative VTE (*n* = 52).

**Fig. 1:**
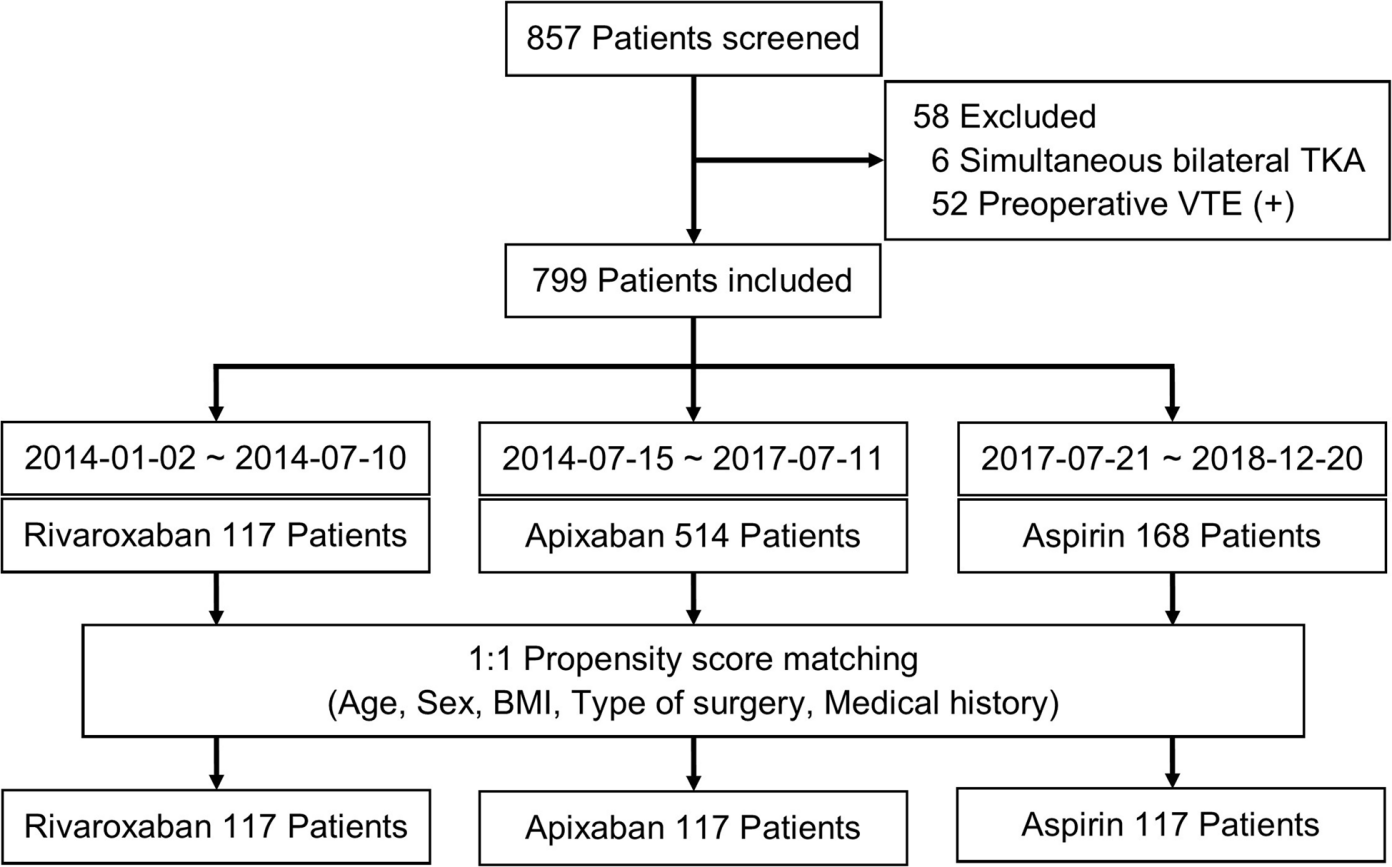
Flowchart of reasons for exclusion and propensity score-matching

Ultimately, 799 patients (83 male and 716 female) were enrolled. Of them, 438 patients underwent unilateral TKA, and 361 patients underwent staged bilateral TKA. Bilateral staged TKA was performed at 1-week intervals; bilateral TKA surgery performed at intervals > 4 weeks was defined as two unilateral TKAs. In all, 168 patients were prescribed aspirin, 117 were given rivaroxaban, and 514 took apixaban (Fig. 1).

To address potential bias and confounding factors, rigorous adjustment was conducted using 1:1 propensity score-matching (caliper 0.2); the matching criteria were age, sex, body mass index (BMI), type of surgery, and medical history. After matching, 117 patients were remained in each agent (Table 1).

**Table 1:** Summary of the demographic characteristics of the study groups

	Total population (*n* = 799)				Propensity-matched population (*n* = 351)			
	Aspirin (*n* = 168)	Rivaroxaban (*n* = 117)	Apixaban (*n* = 514)	*p* value	Aspirin (*n* = 117)	Rivaroxaban (*n* = 117)	Apixaban (*n* = 117)	*p* value
Age (years)	68.7 ± 5.6	75.9 ± 6.0	71.1 ± 6.9	0.001	71.1 ± 4.5	75.9 ± 6.0	76.3 ± 5.9	< 0.001
					71.1 ± 4.5	75.9 ± 6.0		< 0.001
						75.9 ± 6.0	76.3 ± 5.9	0.644
Sex								
Male	19 (11.3)	22 (18.8)	42 (8.2)	0.003	18 (15.4)	22 (18.8)	20 (17.1)	0.786
Female	149 (88.7)	95 (81.2)	472 (91.8)		99 (84.6)	95 (81.2)	97 (82.9)	
Body mass index (kg/m^2^)	27.4 ± 3.8	26.4 ± 3.5	26.7 ± 3.4	0.021	27.1 ± 3.5	26.4 ± 3.5	26.2 ± 3.2	0.126
Type of surgery								
Unilateral	92 (54.8)	80 (68.4)	266 (51.8)	0.005	68 (58.1)	80 (68.4)	74 (63.2)	0.266
Staged bilateral	76 (45.2)	37 (31.6)	248 (48.2)		49 (41.9)	37 (31.6)	43 (36.8)	
Medial history								
HTN	103 (61.3)	81 (69.2)	333 (64.8)	0.387	78 (66.7)	81 (69.2)	83 (70.9)	0.777
DM	32 (19.0)	26 (22.2)	111 (21.6)	0.746	26 (22.2)	26 (22.2)	30 (25.6)	0.775
History of VTE	0 (0.0)	1 (0.9)	1 (0.2)	^a^	0 (0.0)	1 (0.9)	1 (0.9)	^a^
History of CAD	12 (7.1)	7 (6.0)	19 (3.7)	0.151	10 (8.5)	7 (6.0)	7 (6.0)	0.669
Heart valve disease	0 (0.0)	0 (0.0)	3 (0.6)	^a^	0 (0.0)	0 (0.0)	2 (1.7)	^a^
Arrhythmia	3 (1.8)	6 (5.1)	11 (2.1)	^a^	3 (2.6)	6 (5.1)	3 (2.6)	^a^
Malignant disease	13 (7.7)	9 (7.7)	33 (6.4)	0.785	9 (7.7)	9 (7.7)	10 (8.5)	0.962

Values are presented as mean ± standard deviation or number (%). The statistical significance was set at *p* < 0.05

*HTN* hypertension, *DM* diabetes mellitus, *VTE* venous thromboembolism, *CAD* coronary artery disease

^a^The frequency was too low to produce meaningful results

### Routine protocol

Patients were admitted 2 days prior to surgery to check for preoperative VTE via computed tomographic (CT) venography or ultrasonography. Antithrombotic agents commenced on postoperative day 1. Patients undergoing staged bilateral TKA took aspirin between the two operations, and they commenced a designated antithrombotic agent on day 1 after the second operation. The antithrombotic continued until postoperative day 7. All patients were screened for VTE by ultrasonography on postoperative day 6 after unilateral TKA or the second operation of staged bilateral TKA. Because ultrasonography has high sensitivity and specificity, additional tests were performed only in the presence of symptomatic VTE [19]. All patients were followed up at outpatient clinic at 1 month, 3 months, 6 months, and 1 year after surgery. The drug doses were aspirin 300 mg daily, rivaroxaban 10 mg daily, and apixaban 2.5 mg twice daily [13, 20–22].

Other than antithrombotic agents, drugs that could affect thrombotic tendency were not used. In some patients, intra-articularly administered tranexamic acid (IA-TXA) was used, but it it known that IA-TXA does not increase the risk of thromboembolic adverse events [23]. Additionally, all patients wear compression stocking and used intermittent pneumatic compression devices.

### VTE detection

The lower-extremity thrombosis (LET) classification system was used (class I: calf vein thrombosis, class II: popliteal and femoral vein thrombosis, class III: common femoral/iliac vein thrombosis, and class IV: inferior vena caval thrombosis) [24]. If pain, swelling, redness, or warmth was evident, VTE was considered symptomatic.

### Surgical technique

A single surgeon performed all surgeries via an anterior midline skin incision and a standard mid-vastus arthrotomy with tourniquet inflation. The posterior cruciate ligament was resected, tibial extramedullary guide was used and a posterior-stabilized knee prosthesis with a fixed bearing was implanted in all cases. Patellar resurfacing was routine and cement fixation was used in every case. After tourniquet deflation and meticulous bleeding control, a suction drain was inserted and the capsule, vastus muscle fascia, and medial patellar retinaculum were closed. After wound closure with a sterile dressing, tight Jones compression was performed in the extension position.

### Statistical analyses

The results are given as means with standard deviations (SDs). Categorical variables were analyzed using Pearson’s chi-square test and continuous variables (age and BMI) were compared employing Student’s *t* test. Continuous variables subjected to multiple comparisons between agent groups were analyzed using analysis of variance (ANOVA). Propensity scores were estimated via multiple logistic regression analyses including all relevant covariates (age, sex, BMI, type of surgery, and medical history). The antithrombotic groups exhibited 1:1 ratio with calipers equal to 0.2 of the SD of the logistic of the propensity score. Standardized mean differences were estimated to balance all reference covariates before and after matching. All statistical analyses were performed using SPSS ver. 23.0 for Windows (SPSS Inc., Chicago, IL, USA) and RStudio ver. 1.2.5033 for Windows (RStudio Inc., Boston, MA, USA).

## Results

The incidence of postoperative VTE was 15.4% (123/799), the majority of which were calf vein thromboses (98.4%, 121/123). Only one patient developed symptomatic VTE and was prescribed apixaban (an antithrombotic agent). This patient visited the internal medicine clinic and was prescribed apixaban for the next 5 months. And then, treatment concluded without any problem.

To determine risk factors for postoperative VTE, the 799 patients were divided into 676 without VTE and 123 with VTE. It was found that female sex and staged bilateral TKA were significant VTE risk factors with odds ratios of 3.94 and 1.75, respectively (Table 2).

**Table 2:** Demographic characteristics of postoperative venous thromboembolism (VTE)

	Postoperative VTE (−) (*n* = 676)	Postoperative VTE (+) (*n* = 123)	Total (*n* = 799)	RR (95% CI)	*p* value
Age (years)	71.2 ± 7.1	71.9 ± 5.6			0.229
Sex					
Male	79 (95.2)	4 (4.8)	83	3.94 (1.42–11.0)	0.005
Female	597 (83.4)	119 (16.6)	716		
Body mass index (kg/m^2^)	26.7 ± 3.5	27.1 ± 3.4			0.349
Type of surgery					
Unilateral	385 (87.9)	53 (12.1)	438	1.75 (1.19–2.58)	0.004
Staged bilateral	291 (80.6)	70 (19.4)	361		
^a^Previous agents					
No	534 (85.0)	94 (15.0)	628	1.16 (0.74–1.83)	0.522
Yes	142 (83.0)	29 (17.0)	171		

Values are presented as mean ± standard deviation or number (%). The statistical significance was set at *p* < 0.05

*VTE* venous thromboembolism, *RR* relative risk, *CI* confidence interval

^a^Previously prescribed anticoagulant agents for other diseases regardless of total knee arthroplasty (TKA)

After propensity score-matching, no significant differences in the demographic characteristics of the three antithrombotic groups were evident, except for age (Table 1). The incidences of postoperative VTE in the aspirin, rivaroxaban, and apixaban groups were 16.2%, 6.0%, and 17.1% (Table 3), significantly lower in the rivaroxaban group (*p* = 0.02). The majority of VTEs in all three groups were calf vein thromboses. One popliteal VTE developed in the aspirin group, but did not progress to a pulmonary embolism.

**Table 3:** Postoperative venous thromboembolism (VTE) of study groups

	Aspirin (*n* = 117)	Rivaroxaban (*n* = 117)	Apixaban (*n* = 117)	*p* value
Venous thromboembolism, *n* (%)	19 (16.2)	7 (6.0)	20 (17.1)	0.02
VTE classification, *n* (%)				
LET I (calf vein)	18 (94.7)	7 (100.0)	20 (100.0)	
LET II (popliteal/femoral vein)	1 (5.3)	0	0	
LET III (common femoral/iliac vein)	0	0	0	
LET IV (inferior vena cava)	0	0	0	

The statistical significance was set at *p* < 0.05

*VTE* venous thromboembolism, *LET* lower-extremity thrombosis

## Discussion

The incidence of postoperative VTE was 15.4%, principally calf vein thrombosis. Covariate analyses of postoperative VTE frequencies showed that female sex and bilateral staged TKA were VTE risk factors. Of the three antithrombotic agents, rivaroxaban had the lowest incidence of postoperative VTE and majority of VTEs in all three groups were calf vein thromboses.

The incidence of postoperative VTE is 38.8–48.6% if antithrombotic agents are not given after TKA [7, 25]. All subjects in this study were prescribed antithrombotic agents; the postoperative VTE rate was 15.4%. Of the 123 VTE patients, only two were popliteal vein thromboses and the rest were calf vein thromboses.

Various demographics were investigated to determine postoperative VTE risk factors. Statistical analyses focused on age, BMI, type of surgery, medical history, and previous anticoagulant usage. Sex and unilateral or staged bilateral TKA were associated with postoperative VTE. Female patients were 3.69-fold (1.33- to 10.3-fold) more likely than male patients to develop VTE, and those undergoing staged bilateral TKA were 1.68-fold (1.14- to 2.48-fold) more likely to develop VTE than patients undergoing unilateral TKA. Some studies have reported that recurrent venous thromboemboli are more common in male than in female patients, perhaps explained by differences in height or hormonal profiles [26, 27]. Another study found that venous thromboemboli are more common in female patients after TKA [28]. In this study, the female risk was 3.69-fold that of male patients. The majority of VTEs (both sexes) were calf vein thromboses. Surgery and immobilization per se are both major risk factors for VTE [3]. In patients undergoing bilateral staged TKA, the surgical risk doubles and the between-surgery immobilization is also a risk factor. Therefore, it is thought that bilateral staged TKA is a risk factor for VTE.

It was found that age, sex, BMI, and type of surgery significantly differed between the groups receiving aspirin, rivaroxaban, and apixaban. Thus, propensity score-matching was performed. Each group then contained 117 patients who differed only in terms of age. The incidence of VTE was lowest in the rivaroxaban group. Patients in the aspirin group were significantly younger than those in the other two groups. In general, the more advanced the age, the higher the incidence of VTE [29, 30]. The incidence of VTE was lower in the rivaroxaban than the aspirin group although the age of the rivaroxaban group was higher. The rivaroxaban and apixaban groups did not differ in terms of age.

The incidence of postoperative VTE was the lowest in the rivaroxaban group, but the majority of VTEs in all groups were calf vein thromboses associated with low mortality. Only one patient developed symptomatic VTE and none progressed to a pulmonary embolism. Since the purpose of antithrombotic agents is to prevent fatal complications, such as pulmonary embolism, rather than reducing the incidence of VTE, all three agents showed enough efficacy. Moreover, considering that aspirin is inexpensive, aspirin can be considered cost-effective.

This study has some limitations. First, this study has a retrospective design, and confounders could not be controlled. However, propensity score-matching of several preoperative variables was used; thus, bias was limited. Second, ultrasonography was used to screen for postoperative VTE, but both CT venography and ultrasonography were used to screen for preoperative VTE. However, both techniques are very accurate (96.8–97.2%) [19] and it was not considered that there was any device bias. Third, even after propensity score-matching, the groups significantly differed in terms of age. The rivaroxaban group is older than the aspirin group, but age is the risk factor of VTE, so the incidence of VTE is the lowest among the three groups. However, if there are more patients and the ages are matched by propensity score, better results can be obtained.

## Conclusions

In each orally administered antithrombotic agent group, the majority of VTEs were calf vein thromboses associated with low mortaltiy. Although the incidence of postoperative VTE was lowest in the rivaroxaban group, all agents showed enough efficacy as antithrombotic agents. Considering that aspirin is inexpensive, aspirin is a cost-effective option for preventing postoperative VTE.

## Data Availability

The datasets used and/or analyzed during the current study are available from the corresponding author on reasonable request.
